# Supplementary material for: High-coverage allele-resolved single-cell DNA methylation profiling reveals cell lineage, X-inactivation state, and replication dynamics
Source: Nat Commun. 2025 Jul 8;16:6273. doi: 10.1038/s41467-025-61589-1 (PMC12234882; doi:10.1038/s41467-025-61589-1)
Supplement: Supplementary file 1 — Supplementary Information [file 41467_2025_61589_MOESM1_ESM.pdf]

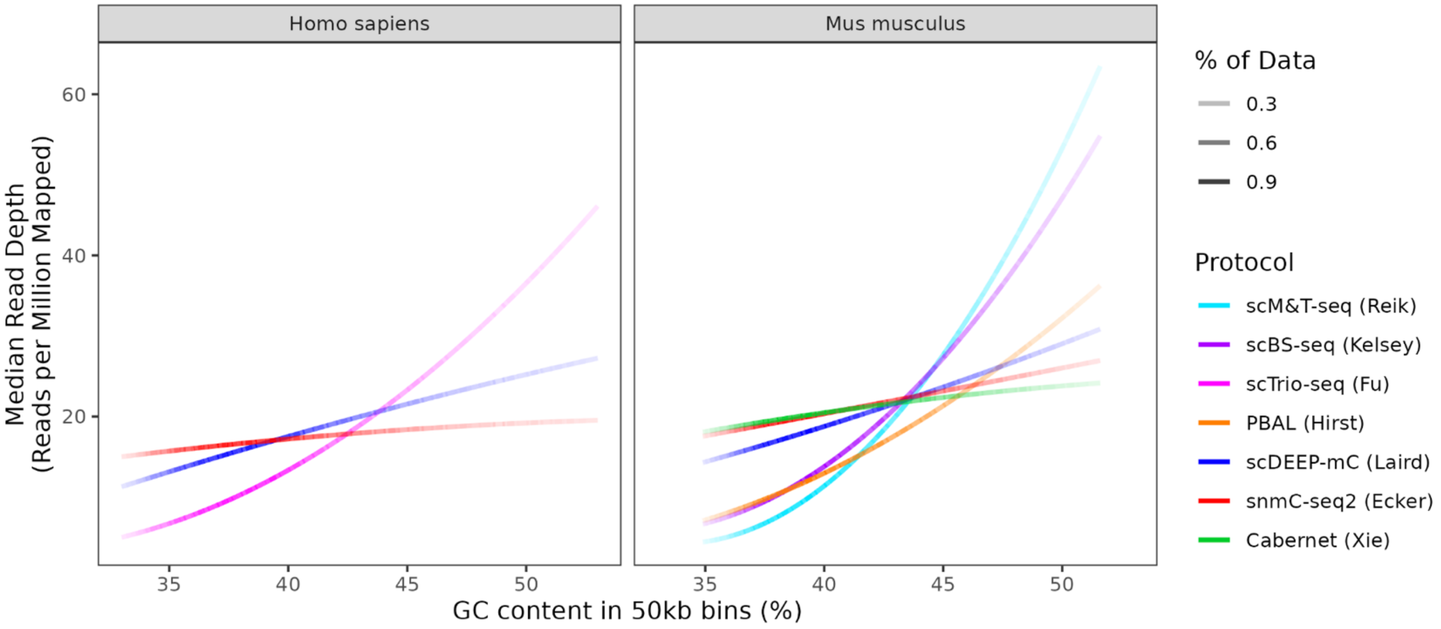

Supplementary Figure 1. GC content bias across all protocols. Normalized read count (reads per million mapped) was tallied for each sequenced cell in 50kb bins. The median normalized read count across all cells and GC content was calculated for each species and bin. Then, modal quantile regression was used to fit a line through this data (binned at 0.1% GC content intervals), representing the most common median read depth at a given GC content for each method. The fraction of data falling into each GC content bin is coded by opacity. Source data are provided as a Source Data file.

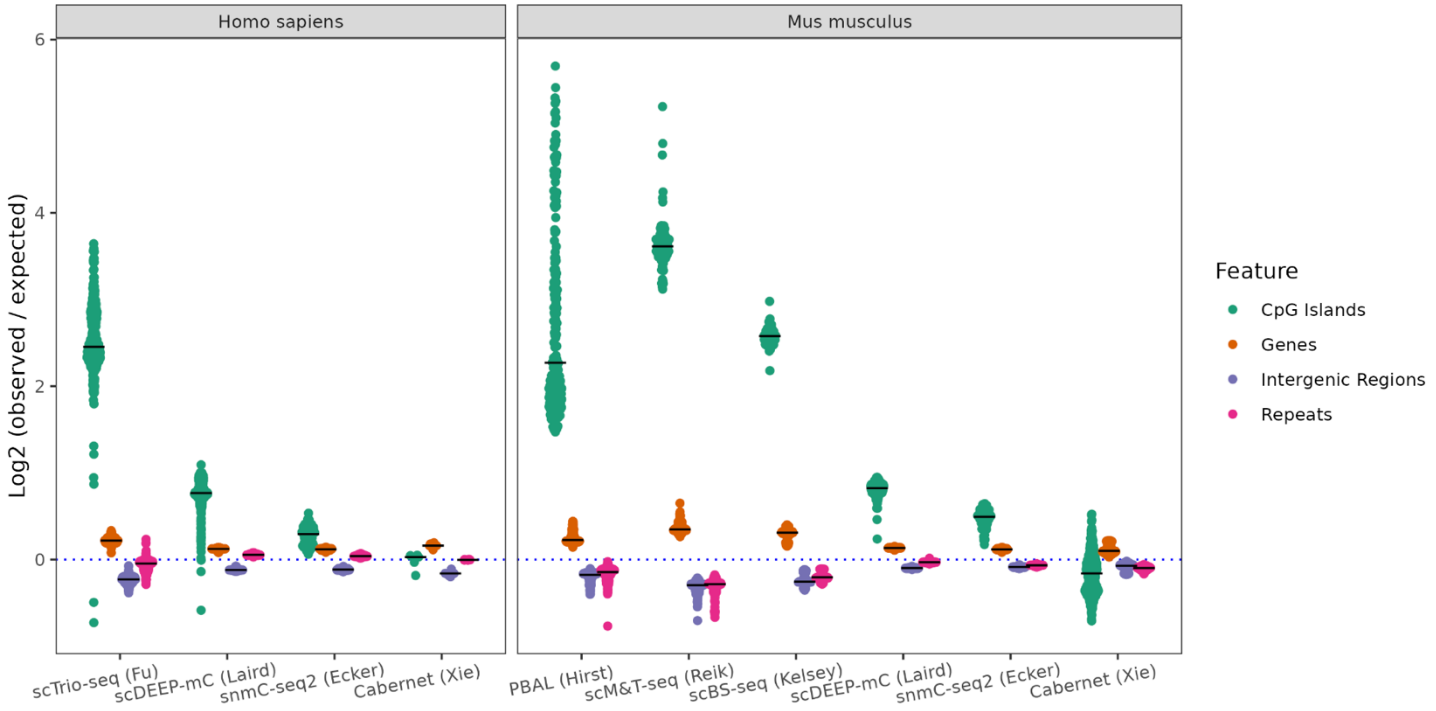

Supplementary Figure 2. Genomic feature representation in various scWGBS libraries. RefSeq gene annotations were downloaded from NCBI. CpG island and RepeatMasker regions were downloaded from UCSC. Intergenic regions were calculated by subtracting the RefSeq annotations from the complete genome. The observed frequency of bases in each region was calculated for each cell (number of bases in feature / number of sequenced bases), and the expected frequency was calculated for each feature and genome (number of bases in feature / number of bases in genome). The median for each region and protocol is shown with a horizontal bar. Data shown from n = 557 cells (human) or n = 793 cells (mouse). Source data are provided as a Source Data file.

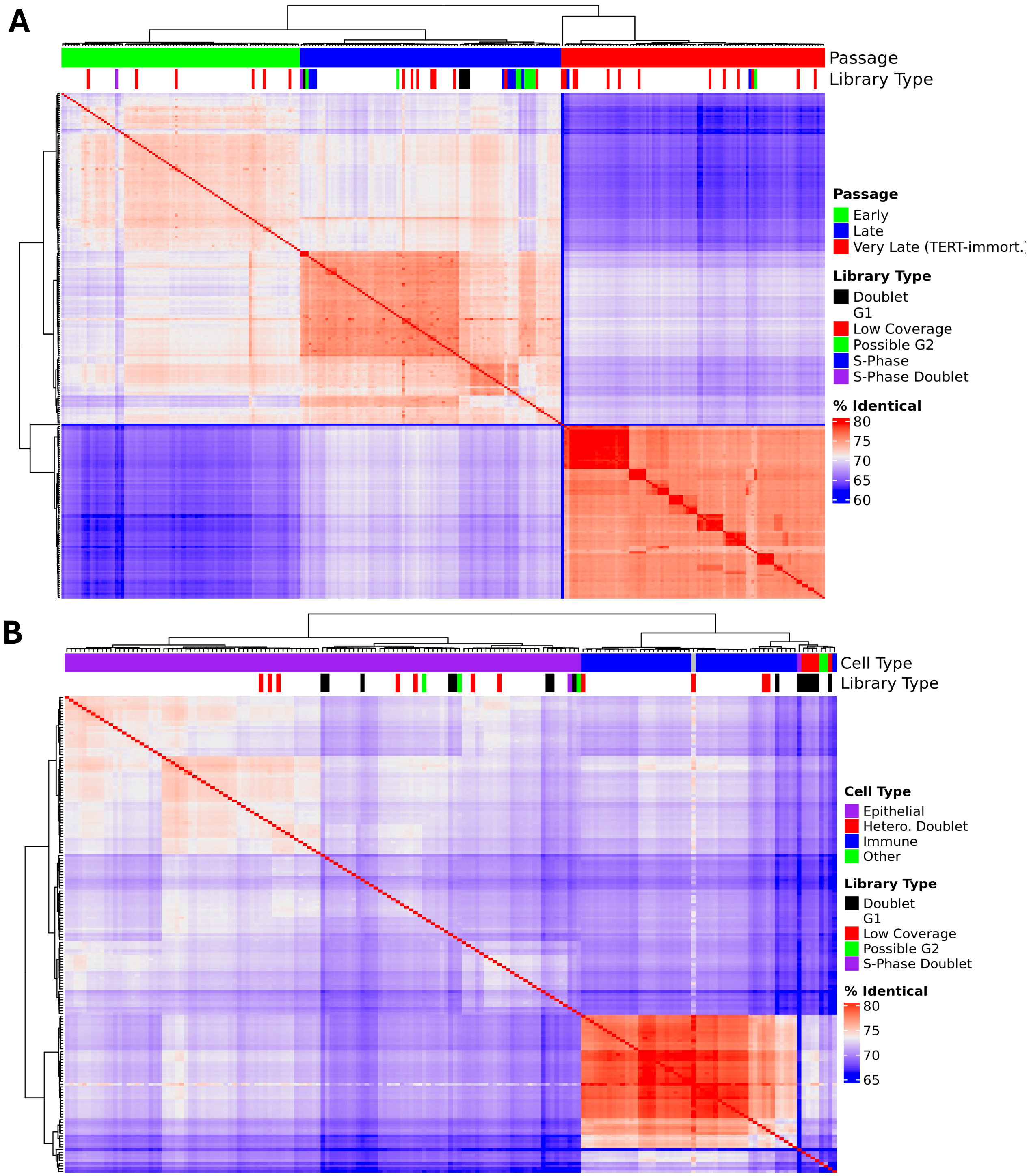

Supplementary Figure 3. Pairwise methylation concordance. Pairwise concordance (number of identical methylation calls / number of shared methylation calls) is shown for all sequenced human fibroblast cells (a) and mouse intestinal cells (b). Concordance values were hierarchically clustered using Ward's algorithm for visualization. Passage level (early, late, or very late), library type, and cell type (inferred as described in the Methods) are shown in the annotations above each heatmap. Data from n = 269 cells (a) or n = 175 cells (b). Source data are provided as a Source Data file.

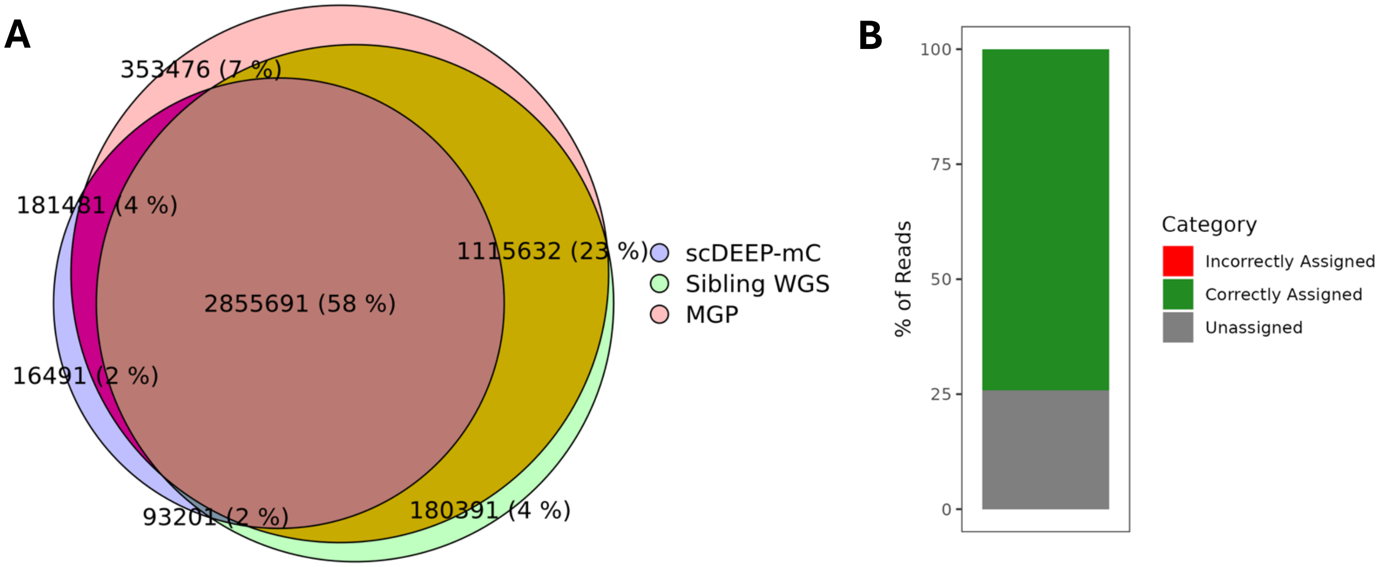

Supplementary Figure 4. Allele-resolved methylation calling performance. A) Euler diagram showing overlap between SNPs discovered in an CB6F1/J mouse using scDEEP-mC data, whole-genome sequencing of 4 littermates, or SNPs from the Mouse Genome Project (MGP). B) 150 million synthetic paired-end WGBS reads were generated from each allele of a synthetic diploid genome containing 3,490,829 heterozygous SNPs using Sherman (https://github.com/FelixKrueger/Sherman) with a CpG conversion rate of 35%, a CpH conversion rate of 99%, and an error rate of 0.2%. These reads were aligned to the reference genome using Biscuit and allele-specific methylation calls were extracted using our pipeline. After assigning reads to alleles, we measured how many reads were assigned to the correct allele, the incorrect allele, or not assigned. Only reads overlapping SNPs are shown in B. Source data are provided as a Source Data file.

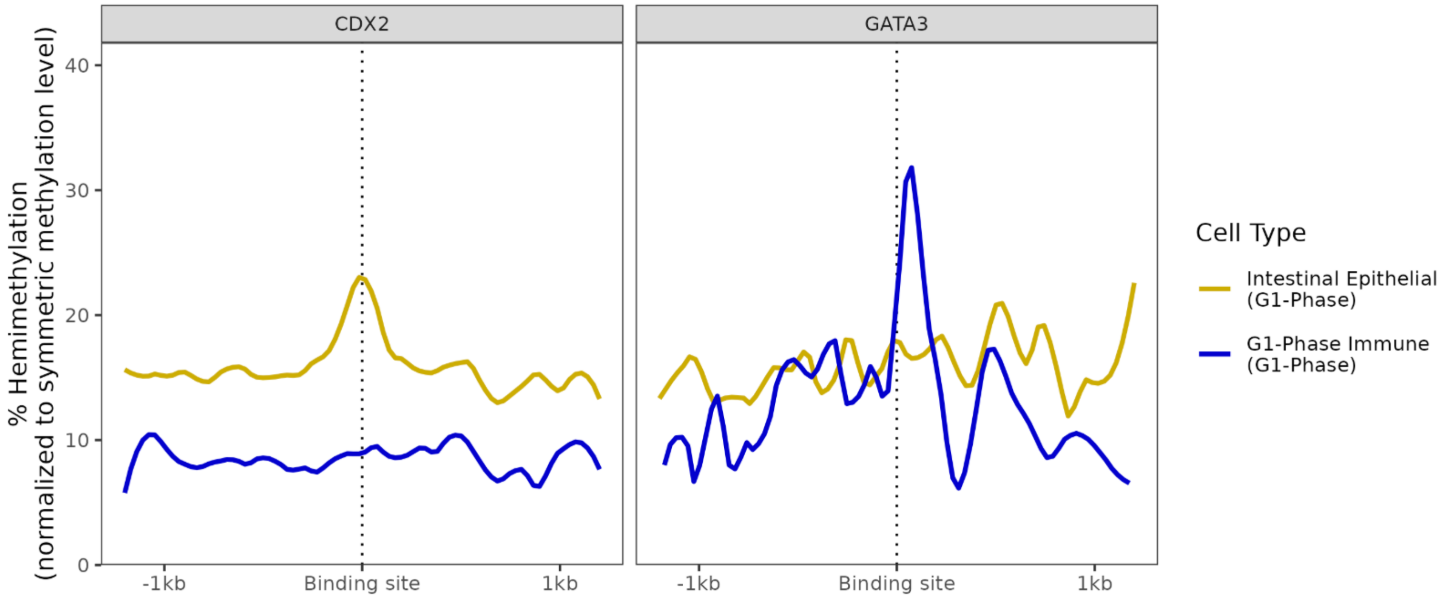

Supplementary Figure 5. Hemi-methylation enrichment around transcription factor binding sites. Hemi-methylation and symmetric methylation rates were computed for each cell type in 25bp bins within 1kb of the transcription factor binding site. A loess fit was used to smooth the data for visualization (span = 0.15). Source data are provided as a Source Data file.

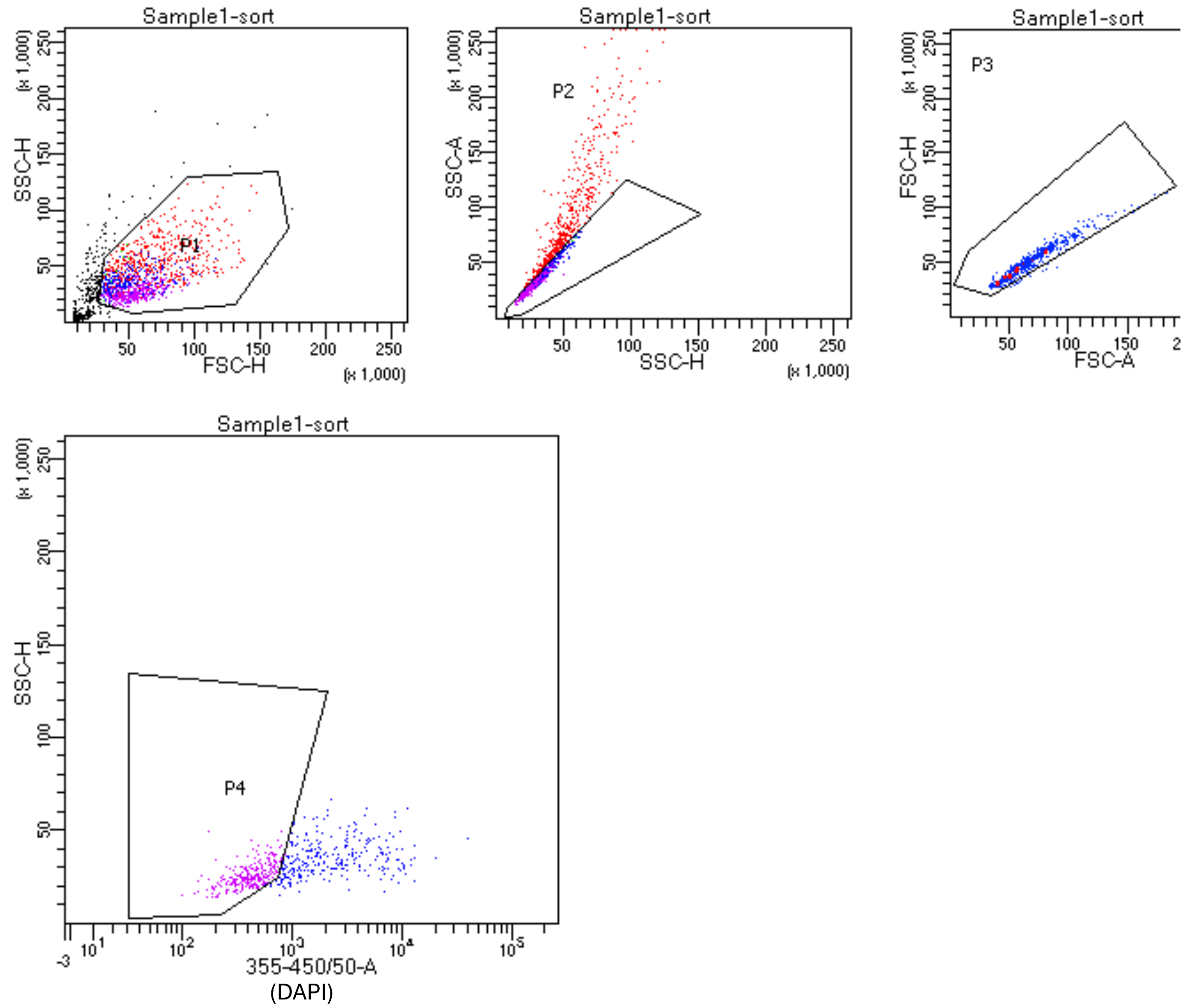

Tube: sort

Population	#Events	%Parent	%Total
All Events	1,666	####	100.0
P1	1,114	66.9	66.9
P2	575	51.6	34.5
P3	566	98.4	34.0
P4	275	48.6	16.5

Supplementary Figure 6. Representative flow sorting gating strategy. Single cells were strictly gated based on forward and side scatter (top panels); live cells were selected based on exclusion of DAPI (middle). Single live cells were sorted from population P4 (bottom).
